# Supplementary figures and images for: RNA m6A methyltransferase activator affects anxiety-related behaviours, monoamines and striatal gene expression in the rat
Source: Acta Neuropsychiatr. 2024 Oct 9;37:e52. doi: 10.1017/neu.2024.36 (PMC13130313; doi:10.1017/neu.2024.36)

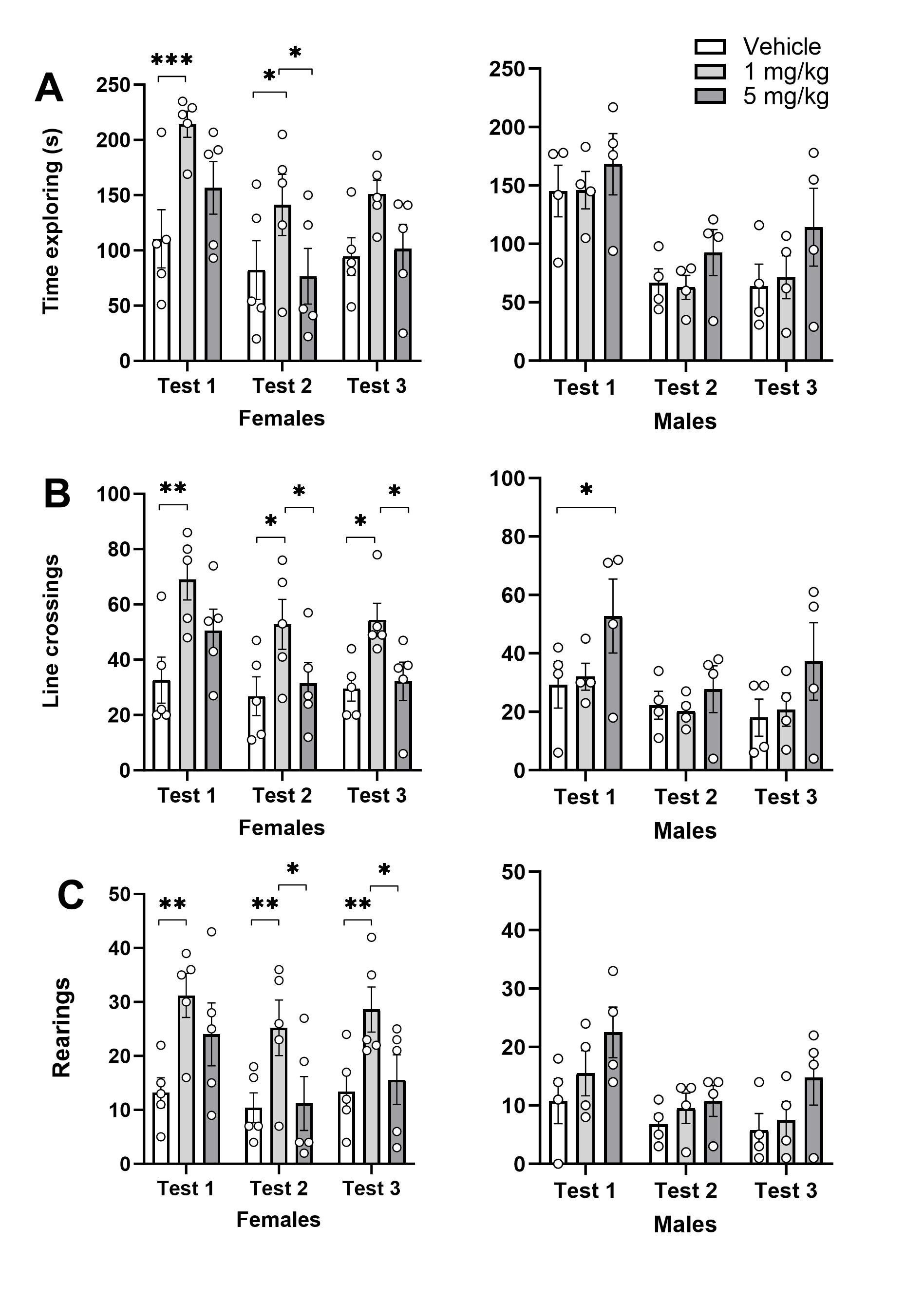

Supplement: Kanarik et al. supplementary material 1 — Kanarik et al. supplementary material [file S092427082400036Xsup001.zip › Supplementary Figure 1.jpg]

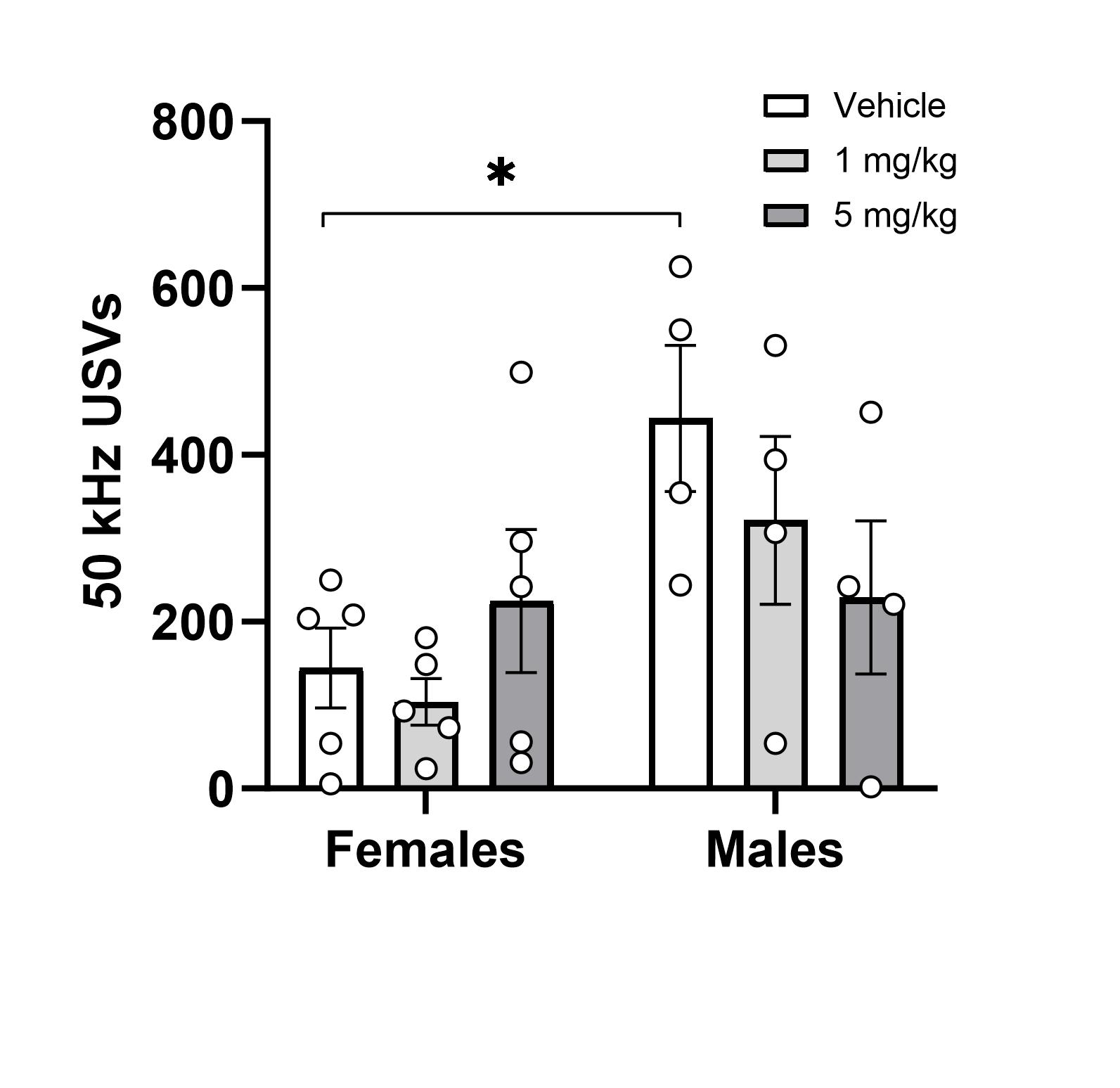

Supplement: Kanarik et al. supplementary material 2 — Kanarik et al. supplementary material [file S092427082400036Xsup002.zip › Supplementary Figure 2.jpg]

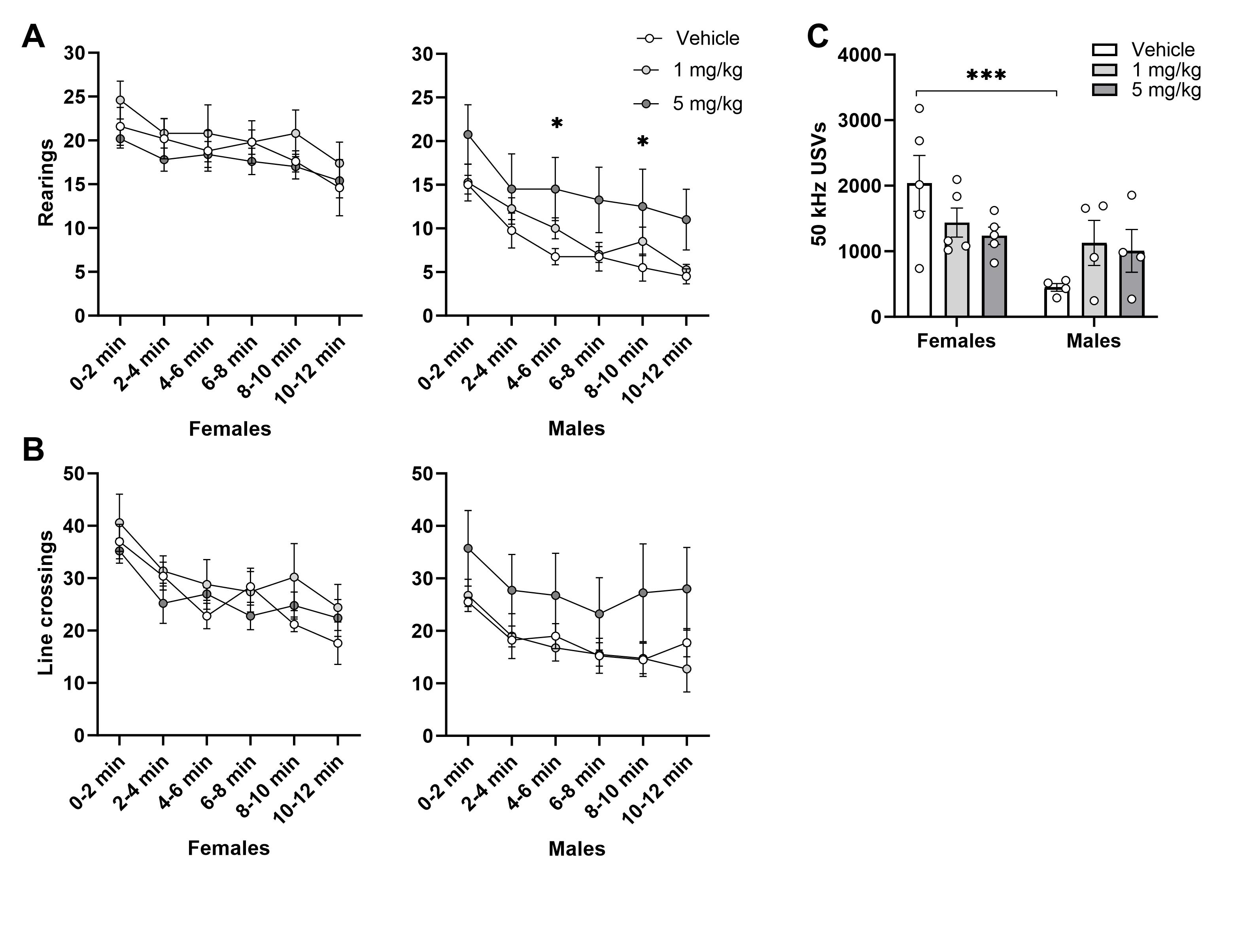

Supplement: Kanarik et al. supplementary material 3 — Kanarik et al. supplementary material [file S092427082400036Xsup003.zip › Supplementary Figure 3.jpg]

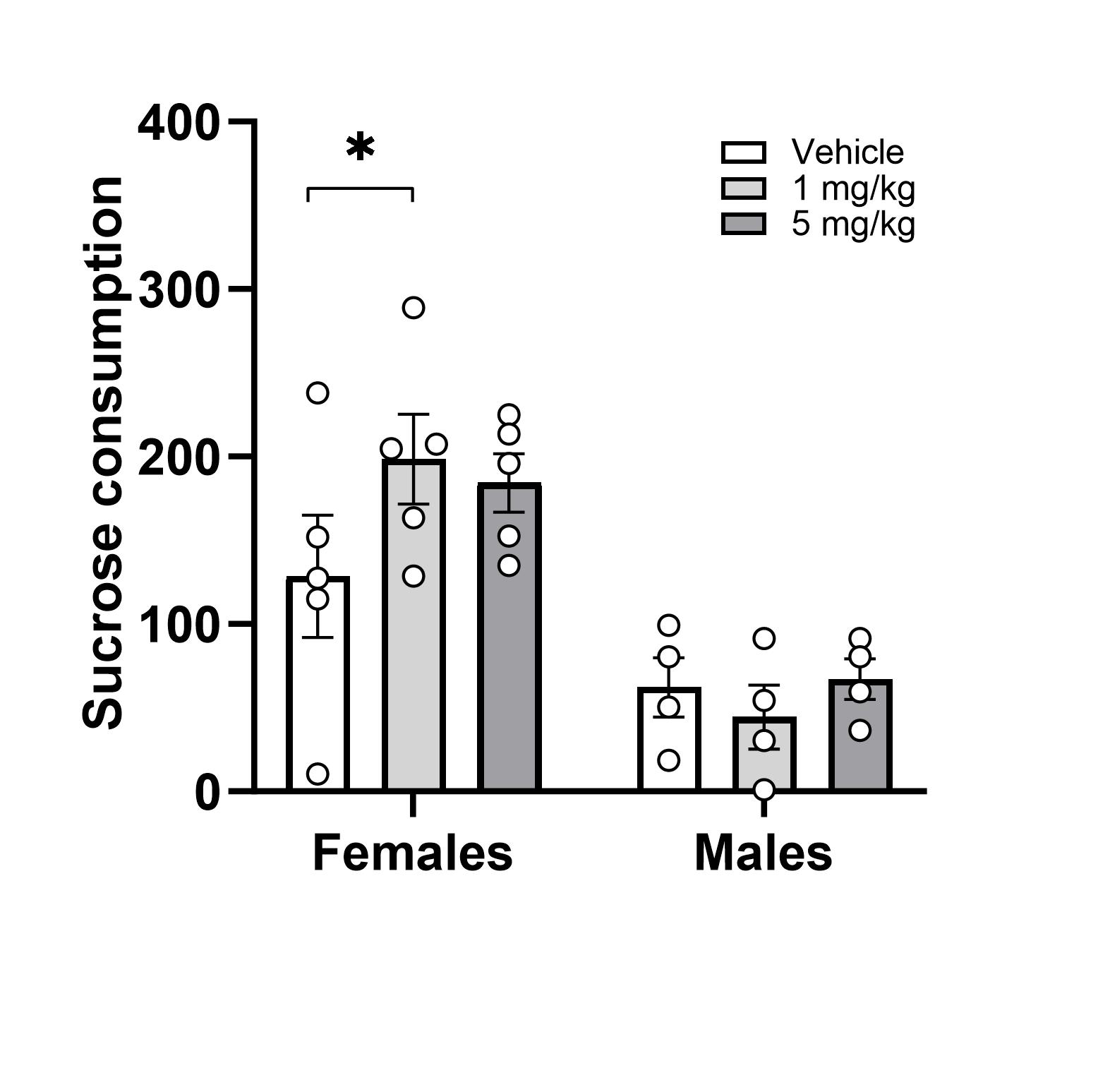

Supplement: Kanarik et al. supplementary material 4 — Kanarik et al. supplementary material [file S092427082400036Xsup004.zip › Supplementary Figure 4.jpg]
